# Liquid–Liquid Phase Separation Primes Spider Silk Proteins for Fiber Formation via a Conditional Sticker Domain

**DOI:** 10.1021/acs.nanolett.3c00773

**Published:** 2023-04-21

**Authors:** Axel Leppert, Gefei Chen, Dilraj Lama, Cagla Sahin, Vaida Railaite, Olga Shilkova, Tina Arndt, Erik G. Marklund, David P. Lane, Anna Rising, Michael Landreh

**Affiliations:** † Department of Microbiology, Tumor and Cell Biology, Karolinska Institutet, S-17165 Solna, Sweden; ‡ Department of Biosciences and Nutrition, Karolinska Institutet, S-14157 Huddinge, Sweden; § Linderstro̷m-Lang Centre for Protein Science, Department of Biology, University of Copenhagen, 2200 Copenhagen, Denmark; ∥ Department of Chemistry − BMC, Uppsala University, S-75123 Uppsala, Sweden; ⊥ Department of Anatomy Physiology and Biochemistry, Swedish University of Agricultural Sciences, 750 07 Uppsala, Sweden; # Department of Cell and Molecular Biology, Uppsala University, S-75124 Uppsala, Sweden

**Keywords:** Phase separation, native mass spectrometry, stickers and spacers-model, functional amyloid

## Abstract

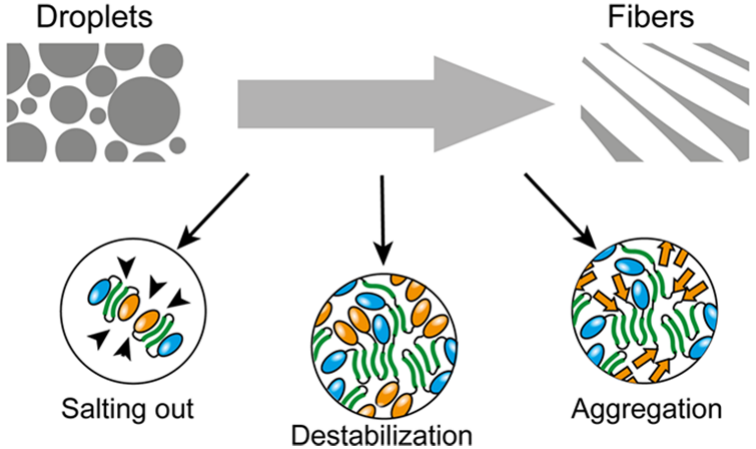

Many protein condensates can convert to fibrillar aggregates, but the underlying mechanisms are unclear. Liquid–liquid phase separation (LLPS) of spider silk proteins, spidroins, suggests a regulatory switch between both states. Here, we combine microscopy and native mass spectrometry to investigate the influence of protein sequence, ions, and regulatory domains on spidroin LLPS. We find that salting out-effects drive LLPS via low-affinity stickers in the repeat domains. Interestingly, conditions that enable LLPS simultaneously cause dissociation of the dimeric C-terminal domain (CTD), priming it for aggregation. Since the CTD enhances LLPS of spidroins but is also required for their conversion into amyloid-like fibers, we expand the stickers and spacers-model of phase separation with the concept of folded domains as conditional stickers that represent regulatory units.

Proteins, despite being only a few nanometers in size, can form macromolecular structures that range from micrometers to centimeters. Some proteins form highly ordered amyloid fibrils via β-sheet aggregation, but also highly dynamic droplets via liquid–liquid phase separation (LLPS). Fibrils are composed of complementary steric zippers whereas LLPS is driven by stickers, pairwise interactions between individual residues or between folded domains, and spacers, flexible connections that contribute to the liquid-like properties of the assemblies.^1,2^ LLPS is important for many cellular processes, such as the formation of membrane-less organelles and the regulation of gene expression. Here, fibrillar structures are considered aberrant states associated with neurodegeneration and cancer that arise from slow maturation of droplets into aggregates.^3^

In case of spider silk proteins, spidroins, droplets and fibers both represent functional states of the same protein. Spidroins can be stored as liquid droplets that can rapidly be converted into fibers (Figure 1a).^4−6^ Factors that control this conversion are acidification, shear force, and changes in ion concentrations, most notably phosphate and bicarbonate.^7,8^ Although the actual ion concentrations in spider glands are not completely known, their changes suggest a crucial role for fiber formation. Mini-spidroins containing 1–6 disordered repeat domains readily undergo LLPS *in vitro* at phosphate concentrations around 0.5 M but can be spun into tough fibers.^4,9,10^ The distinctive ability of spidroins to convert from one state to another prompted us to clarify the mechanistic relationship between LLPS and fiber formation. For this purpose, we turned to a chimeric mini-spidroin (NT2RepCT) composed of two repeat sequences and the folded N- and C-terminal domains (NTD and CTD), and the isolated dimeric CTD.^8^

**Figure 1:**
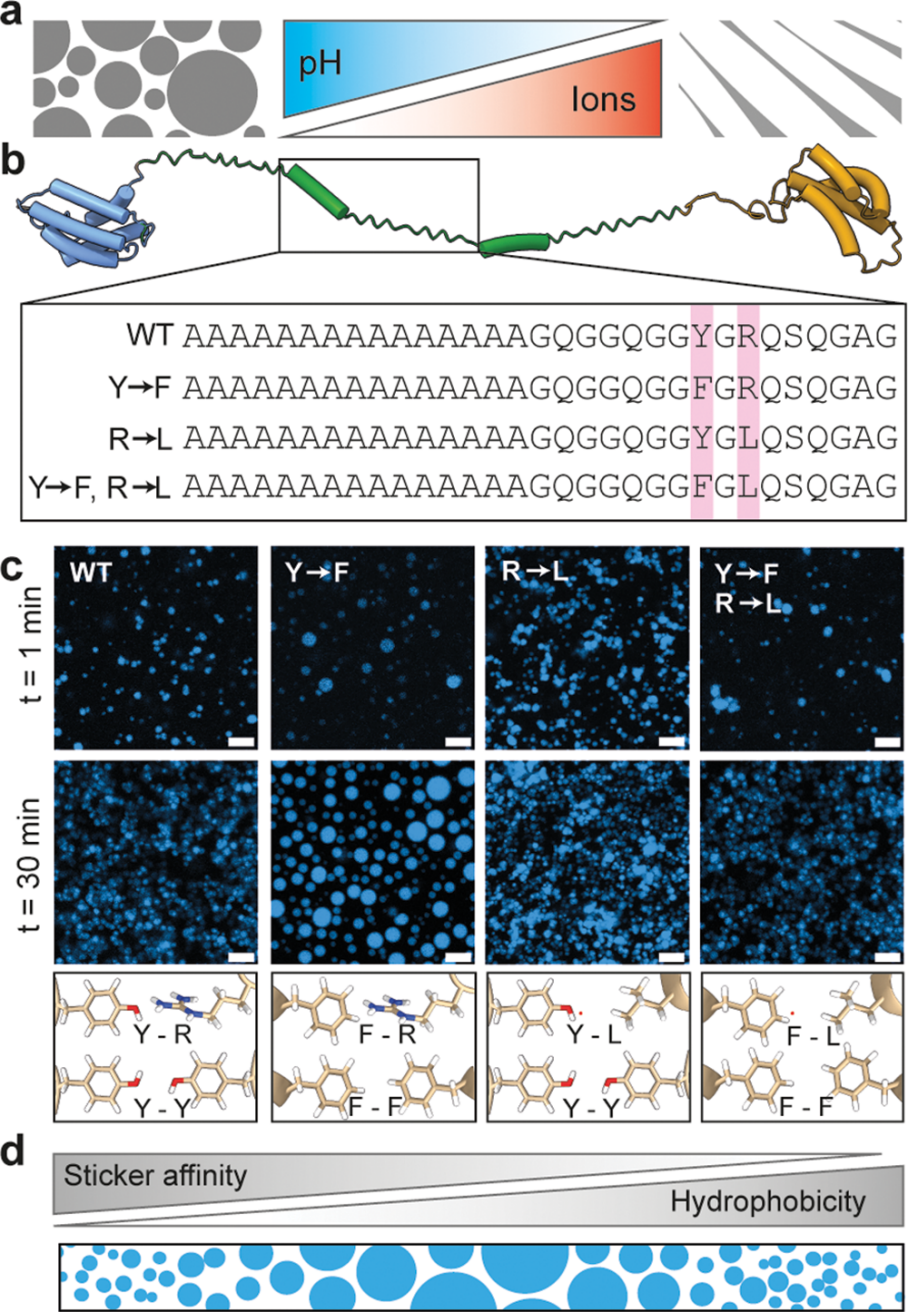
Sequence dependence of NT2RepCT LLPS: (a) Conversion of spidroins from droplets to fibers is accompanied by a decrease in pH and an increase in ion concentration, predominantly phosphate and bicarbonate. (b) Structure of the NT2RepCT mini-spidroin, with NTD (blue) and CTD (yellow), as well as two repeat regions (green) with the sequence of the first repeat of WT, Y to F, and Y to F, R to L shown below. (c) Fluorescence microscopy of all three variants in 0.5 M potassium phosphate shows increased droplet size and fluidity of the Y to F variant. Scale bars are 10 μM. (d) Schematic view of high-affinity stickers and hydrophobic residues illustrates how the balance between sticker affinity and hydrophobicity can control spidroin LLPS.

As first step, we considered the sequence of the repeat domain. Low-complexity sequences are considered hallmarks of phase separating-proteins, with tyrosine, phenylalanine, and arginine stickers engaging in π–π and π–cation interactions.^11,12^ The repeat region of NT2RepCT contains one arginine and two tyrosine in total and we exchanged either tyrosine to phenylalanine (YF),^13^ arginine to leucine (RL), or tyrosine to phenylalanine and arginine to leucine (YFRL) (Figure 1b) and monitored LLPS using fluorescence microscopy with the DroProbe reagent.^14^ All four variants formed droplets immediately after dilution in 0.5 M potassium phosphate, suggesting that LLPS is not solely dependent on these residues. Removing the CTD resulted in fewer droplets, whereas removing the NTD did not affect LLPS (Figure S1).^4^ NT2RepCT^WT^, NT2RepCT^RL^, and NT2RepCT^YFRL^ droplets remained small (<5 μm) and assembled into clusters within 30 min. The Y to F variant (NT2RepCT^YF^), on the other hand, formed larger droplets (>10 μm) that continued to fuse after 30 min (Figure S1, Movie S1). We speculate that the larger droplet size NT2RepCT^YF^ indicates increased fluidity compared to the NT2RepCT^WT^, NT2RepCT^RL^, and NT2RepCT^YFRL^ droplets, which stick together instead of fusing. These observations indicate that the balance between sticker affinity and hydrophobicity likely determines the size and fluidity of the droplets. Interestingly, fibers produced with NT2RepCT^YF^ through biomimetic spinning showed increased supercontraction but otherwise similar mechanical properties as WT fibers.^13^ A recent NMR study of the effect of LLPS on the structures of spidroin fibers revealed that tyrosine residues, which interact during phase separation, remain aligned in the fiber.^15^ These observations indicate that some droplet and fiber properties are related, opening avenues for the rational design of LLPS-competent, fibril-forming proteins.

Recently, it was shown that kosmotropic ions induce a compact conformation of the repeat domains of spidroins.^10,16^ To find out whether salt-induced conformational changes enable LLPS, we turned to native ion mobility mass spectrometry (IMMS). In IMMS, the gentle transfer of protein complexes into the vacuum region of the mass spectrometer preserves aspects of their solution structures. Measuring the time it takes the ionized complexes to traverse a gas-filled drift cell informs about their conformations, and mass measurements about their oligomeric states.^17^ Importantly, IMMS can capture LLPS-related conformational changes in proteins.^18,19^ To be able to probe LLPS with IMMS, we first tested phase separation of NT2RepCT^YF^ in MS-compatible buffers ammonium citrate, ammonium acetate, and ammonium bicarbonate (Figure 2a). Monitoring initial droplet formation (at *t* = 1 min), we found that citrate induces LLPS at concentrations around 0.5 M, comparable to potassium phosphate, as reported previously.^4^ Ammonium acetate required a concentration of 1 M for LLPS, and carbonate induced droplet formation at a concentration of 2 M. The four anions tested here thus induce LLPS following their order in the Hofmeister series (Figure 2a).

**Figure 2:**
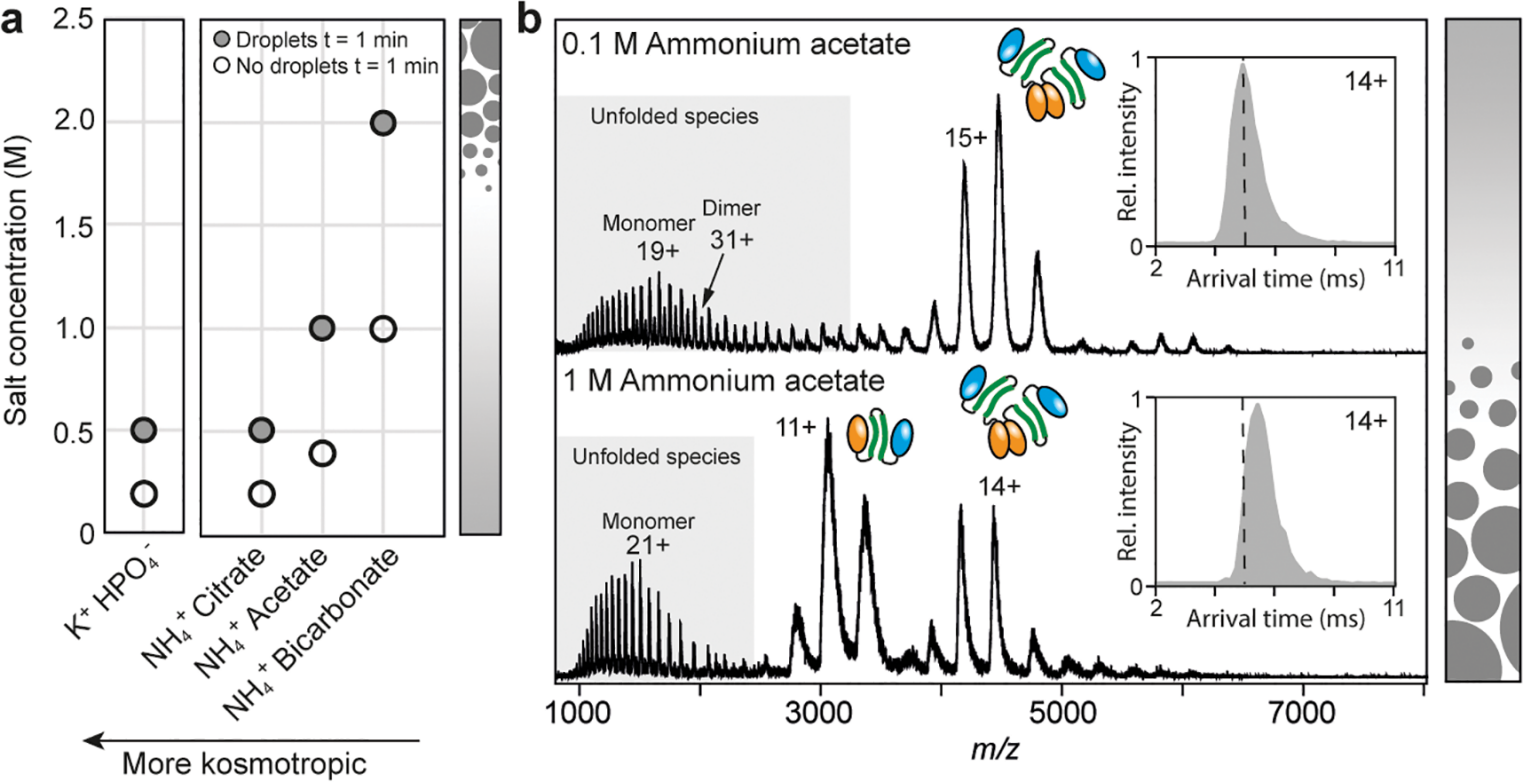
Kosmotropic ions promote LLPS and destabilize the dimeric state of NT2RepCT^YF^. (a) NT2RepCT^YF^ droplet formation shows a strict dependence on salt concentration, requiring higher concentrations of less kosmotropic ions. Open circles show the highest concentration at which almost no droplets were observed directly upon dilution in the respective buffer. (b) Native IMMS of NT2RepCT^YF^ below (upper panel) and above (lower panel) ammonium acetate concentrations that induce LLPS. Compared to 0.1 M ammonium acetate, we find an increase in lowly charged monomers around the 11+ ion, and a decrease in compact as well as in highly charged dimers (around the 14+ and 31+ charge states, respectively) In 1 M ammonium acetate. Inserts: Arrival time distributions for the 14+ NT2RepCT^YF^ dimer reveal a slight increase in drift time at high ammonium acetate concentrations. Dashed lines indicate the arrival time centroid of the native protein in 0.1 M ammonium acetate.

Next, we subjected NT2RepCT^YF^ to IMMS analysis in the three buffer systems (Figure 2b). Unfortunately, we were not able to detect the protein in 0.5 M citrate because the buffer interferes with ionization at this concentration. In 0.1 M ammonium acetate, all NT2RepCT variants gave virtually identical mass spectra (Figure S2). Upon increasing the ammonium acetate concentration from 0.1 to 1 M, we found a pronounced increase in signals corresponding to compact monomers with a narrow charge state distribution centered around the 11+ ion. At 0.3 M ammonium acetate, just below the onset of LLPS, we observe an intermediate regime, with a minor population of compact monomers (Figure S2). Turning to ion mobility measurements, the arrival times for the intact dimer were increased in 1 M ammonium acetate, indicating loosening of the structure (Figure 2b, inset, Figure S2). Next, we analyzed the protein in ammonium bicarbonate. Unlike spectra recorded for ammonium acetate, spectra in the presence of ammonium bicarbonate show a complete loss of NT2RepCT^YF^ dimers even at a buffer concentration of 0.1 M, leaving only highly charged monomers (Figure S2). When the concentration was increased to 1 M, a second monomer population appeared around the 14+ charge state, but still with significantly higher charges than in ammonium acetate (Figure S2). Bicarbonate has a well-known ability to cause partial unfolding of proteins during ionization,^20^ however, the pronounced effect on NT2RepCT^YF^ even under gentle MS conditions suggests that the protein is particularly sensitive to bicarbonate-induced destabilization. Taken together, we conclude that high salt concentrations promote LLPS according to the Hofmeister series but appear to destabilize the native NT2RepCT^YF^ dimer.

Spidroins form constitutive dimers via their CTD, an α-helical 13-kDa domain which includes several amyloidogenic segments and rapidly assembles into fibrillar aggregates when exposed to low pH. Aggregation is mediated by protonation of a conserved charge cluster in the folded protein and triggers fiber assembly.^8,21^ Considering the decrease in intact dimers at high ammonium acetate concentrations, we asked whether the observed increase in monomers is controlled by the CTD. We therefore analyzed the full-length CTD from minor ampullate spidroin 1 (MiSp1) composed of the all-helical core and its nonrepetitive N-terminal 48-residue linker. Such linker sequences are present in all CTDs and have been found to be disordered by NMR spectroscopy.^8,22^ Native MS of the CTD in 0.1 M ammonium acetate at pH 8 shows exclusively dimeric protein, in agreement with the NMR structure (Figure 3a). However, increasing the buffer concentration to 1 M, which induces LLPS of NT2RepCT, resulted in dissociation of the dimer and the appearance of lowly charged, as well as some highly charged, monomers. The CTD spectra thus perfectly recapitulate the effect of ammonium acetate on NT2RepCT.

**Figure 3:**
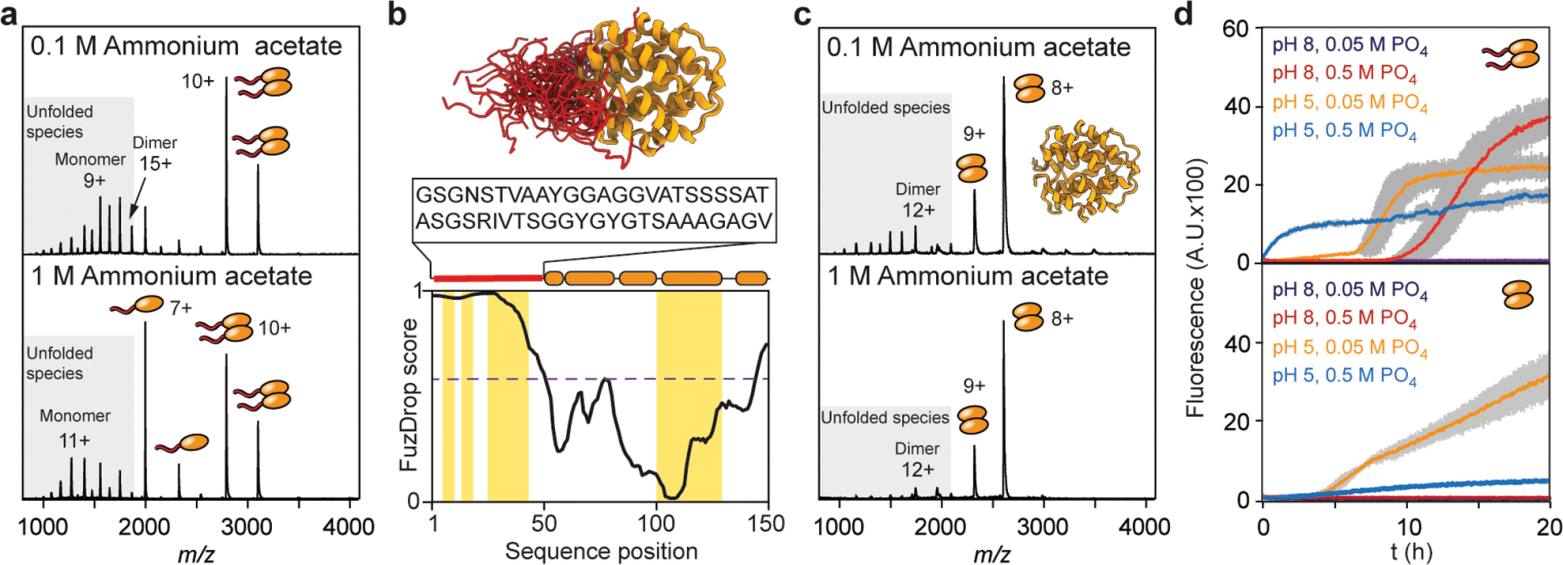
Disordered linker primes the CTD for assembly during LLPS. (a) Native mass spectra show dissociation of the dimeric CTD when the ammonium acetate concentration is raised from 0.1 to 1 M. (b) Overlay of the top 20 NMR structures of the MiSp1 CTD dimer (PDB ID 2MFZ) show part of the disordered linker (red) and the folded core (orange). The linker is rich in residues associated with LLPS and is predicted by the FuzDrop server to undergo LLPS, with the 0.6 threshold shown as dashed line. Yellow boxes denote regions with high propensity for β-sheet aggregation predicted by Aggrescan3D. (c) Truncated CTD without linker domain that is exclusively dimeric in native MS and does not dissociate in response to increased ammonium acetate concentration. (d) Top: ThT fluorescence curves for the full-length CTD in 0.05 or 0.5 M potassium phosphate buffer at pH 8 and 5. Low pH and high phosphate concentration causes lag-free formation of ThT-positive aggregates (blue curve). Bottom: ThT fluorescence curves for the truncated CTD show no aggregation at pH 8, and inhibition of aggregation by 0.5 M phosphate at pH 5 (blue curve). Curves are averages of *n* = 5 repeats., error bars indicate standard deviation.

To find out what part of the CTD mediates the sensitivity of the dimer to LLPS-promoting conditions, we considered the sequence of the linker. Twenty-six of its 48 residues are G, S, or Y (Figure 3b), which are enriched in low-complexity sequences that undergo LLPS.^12^ Consequently, the linker is identified as an LLPS-forming sequence by the FuzDrop server.^23^ To test whether the linker is the element that responds to changes in ammonium acetate concentrations, we produced a truncated CTD lacking the linker. Native MS shows that the truncated CTD remains dimeric regardless of the ammonium acetate concentration (Figure 3c) and, unlike the full-length CTD, remained soluble at high phosphate concentrations (Figure S3), We conclude that the linker disrupts CTD dimerization under high salt conditions that also promote LLPS.

Interestingly, the presence of the CTD is not only required for the conversion into fibers, but also strongly promotes LLPS of spidroins (Figure S1).^4^ To test whether pH and ion concentration indeed act separately on the CTD, we recorded mass spectra of full-length and truncated CTDs at pH 5. We found that both variants exhibit a similar shift to higher charge states, indicating partial unfolding, but remain mostly dimeric (Figure 3). Our observations thus suggest the existence of two separate sensors that affect the structure of the CTD: a conserved cluster of charged residues (Figure S3) which unfolds the CTD in response to low pH, and a linker which dissociates the dimer in response to high ion concentrations.

The ability of the CTD to separately sense changes in pH and ion concentration raises the question of how both processes contribute to the generation of silk fibers. We therefore analyzed the aggregation of the CTD using the β-sheet-specific dye Thioflavin T (ThT) at low and high phosphate concentrations as well as at low and high pH (Figure 3d). At low phosphate concentration, pH 8, the CTD showed no change in ThT fluorescence over several hours, indicating that the protein does not convert to fibrils. Lowering the pH to 5 resulted in a strong increase in fluorescence after approximately 5 h, in good agreement with the known pH dependence of CTD aggregation.^8^ Interestingly, raising the phosphate concentration to 0.5 M induced aggregation at pH 8, albeit with a longer lag time of 10 h. Combining low pH and high phosphate concentration results in a lag time-free increase in ThT fluorescence, suggesting that the protein assembles with a drastically shorter nucleation phase (Figure 3d). Strikingly, the truncated CTD showed no ThT fluorescence at pH 8 regardless of phosphate concentration. Lowering the pH to 5 induced the formation of ThT-positive aggregates at low phosphate concentration, however, raising the phosphate concentration inhibited β-sheet aggregation again. We speculate that the low surface potential of the truncated CTD leads to stabilization at high salt concentrations (Figure S3). In presence of the linker, kosmotropic ions cause assembly of the folded CTDs into aggregation-competent oligomers. Lowering the pH then destabilizes the domain, driving fibrillation. To understand how the linker can sense kosmotropic salts, we performed all-atom MD simulations of four copies of the 23 residues located N-terminally of the folded CTD, which are predicted by AggregScan3D^24^ to contain a large amyloidogenic stretch (Figure 3b), at high and low phosphate concentrations. We found that the linker remains disordered in all conditions. However, in 0.5 M phosphate, the linkers associate into small oligomers, as seen by the decreased distance between the centers of mass of the peptides (Figure S3). Close inspection of the simulated structures suggests that association is driven by increased hydrophobic interactions as well as stacking of tyrosine residues (Figure S3).

Together, our data suggest a direct connection between LLPS and aggregation (Figure 4). Salting out with kosmotropic phosphate ions induces LLPS by compacting the repeat domains, promoting contacts between tyrosine and arginine stickers, while simultaneously increasing aggregation propensity of the CTD. We speculate that the linker engages in similar interactions as the repeat region during LLPS, and in this manner promotes the association of multiple CTDs, which is accompanied by dimer dissociation. The linker may thus help to concentrate CTDs inside the spidroin droplet to enhance fiber nucleation (Figure 4, insert). Notably, NT2RepCT droplets formed at high potassium phosphate concentrations at pH 8 did not convert into fibrillar aggregates within the same time frame as the isolated CTD, which indicates that LLPS can suppress CTD aggregation to some extent. If, however, the fold of the CTD is additionally destabilized by protonation of the central charge cluster, previously locked amyloidogenic regions may become available for self-association, resulting in fiber assembly. In this sense, LLPS appears to prime the spidroins for silk assembly by aligning aggregation-prone sequences prior to fiber formation.

**Figure 4:**
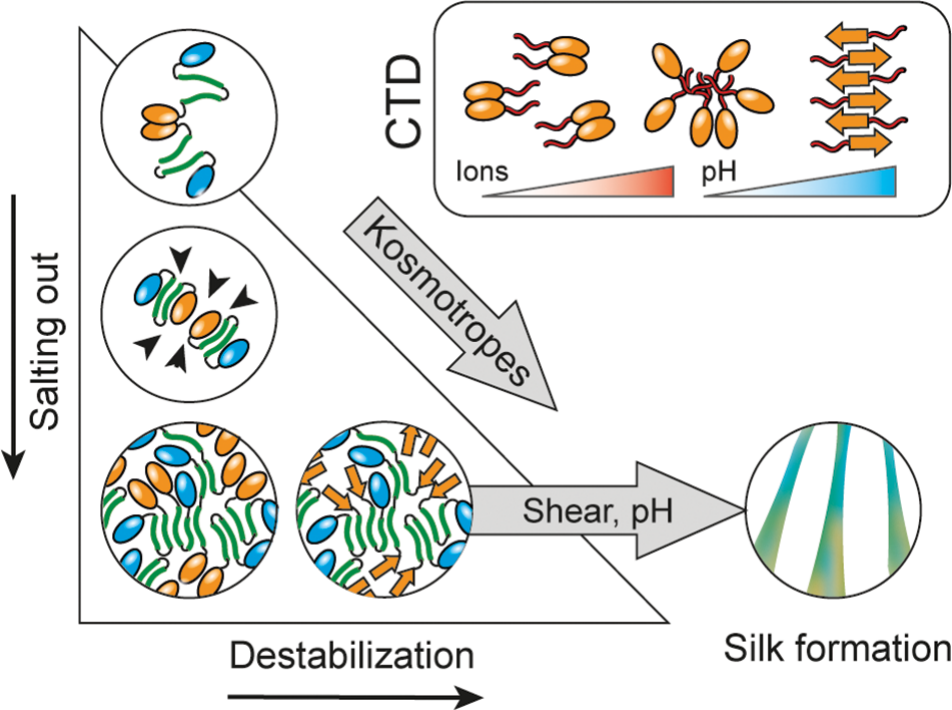
Role of LLPS in spider silk assembly. Kosmotropic ions promote liquid–liquid phase separation through a salting out-mechanism and simultaneously destabilize the CTD dimer by acting on its linker region (insert). Lower pH then triggers full CTD unfolding and aggregation. Both processes this generate droplets of aggregation-competent spidroins. Shear force, which is not studied here, is likely to then mediate the final assembly into fibers.

In the context of the stickers and spacers-model, the fact that the CTD is required for droplet and fiber formation has an interesting implication: On one hand, the CTD can be considered a folded sticker that mediates LLPS. On the other hand, its destabilization drives the conversion of droplets to fibers by nucleating β-sheet aggregation upon external stimuli. We thus propose that the CTD is a conditional sticker regulating the conversion of liquid droplets to solid fibers. Folded domains whose structures change in response to environmental cues offer a simple means to promote or abolish phase separation in specific biological processes.

## Supporting Information Available

DroProbe images of LLPS and end points of the NT2RepCT variants; LLPS and native MS of NT2RepCT variants; structural features of the CTD (PDF)
Light microscopy of NT2RepCT^YF^ droplets after 30 min in 0.5 M potassium phosphate (AVI)

## Supplementary Material

nl3c00773_si_001.pdf

nl3c00773_si_002.avi
