# Supplementary material for: Liquid–Liquid Phase Separation Primes Spider Silk Proteins for Fiber Formation via a Conditional Sticker Domain
Source: Nano Lett. 2023 Apr 21;23(12):5836–41. doi: 10.1021/acs.nanolett.3c00773 (PMC10311596; doi:10.1021/acs.nanolett.3c00773)
Supplement: Supplementary file 1 — nl3c00773_si_001.pdf [file nl3c00773_si_001.pdf]

Liquid-Liquid Phase Separation Primes Spider Silk Proteins for Fiber Formation *via* a Conditional Sticker Domain

Axel Leppert,[1] Gefei Chen,[2] Dilraj Lama,[1] Cagla Sahin,[1,3] Vaida Railaite,[1] Olga Shilkova,[2] Tina Arndt,[2] Erik G. Marklund,[4] David P. Lane,[1] Anna Rising,[2,5,*] and Michael Landreh [1,6,*]

---

[1] Department of Microbiology, Tumor and Cell Biology, Karolinska Institutet, S-17165 Solna, Sweden

[2] Department of Biosciences and Nutrition, Karolinska Institutet, S-14157 Huddinge, Sweden

[3] Linderstrøm-Lang Centre for Protein Science, Department of Biology, University of Copenhagen, 2200 Copenhagen, Denmark

[4] Department of Chemistry – BMC, Uppsala University, S-75123 Uppsala, Sweden

[5] Department of Anatomy Physiology and Biochemistry, Swedish University of Agricultural Sciences, 750 07 Uppsala, Sweden

[6] Department of Cell and Molecular Biology, Uppsala University, S-75124 Uppsala, Sweden

* E-mail: Michael.Landreh@icm.uu.se or Anna.Rising@ki.se

Experimental

All reagents were purchased from Sigma-Aldrich if not stated otherwise.

Protein sequences

CTD_FL

```
        10         20         30         40         50         60
GSGNSTVAAY GGAGGVATSS SSATASGSRI VTSGGYGYGT SAAAGAGVAA GSYAGAVNRL

        70         80         90        100        110        120
SSAEAASRVS SNIAAIASGG ASALPSVISN IYSGVVASGV SSNEALIQAL LELLSALVHV

       130        140        150
LSSASIGNVS SVGVDSTLNV VQDSVGQYVG
```

CTD_NL

```
        10         20         30         40         50         60
GSAAGAGVAA GSYAGAVNRL SSAEAASRVS SNIAAIASGG ASALPSVISN IYSGVVASGV

        70         80         90        100        110
SSNEALIQAL LELLSALVHV LSSASIGNVS SVGVDSTLNV VQDSVGQYVG
```

$NT2RepCT^{WT}$

```
        10         20         30         40         50         60
MGHHHHHHMS HTTPWTNPGL AENFMNSFMQ GLSSMPGFTA SQLDDMSTIA QSMVQSIQSL

        70         80         90        100        110        120
AAQGRTSPNK LQALNMAFAS SMAEIAASEE GGGSLSTKTS SIASAMSNAF LQTTGVVNQP

       130        140        150        160        170        180
FINEITQLVS MFAQAGMNDV SAGNSGRGQG GYGQGSGGNA AAAAAAAAAA AAAAGQGGQG

       190        200        210        220        230        240
GYGRQSQGAG SAAAAAAAAA AAAAAGSGQG GYGGQGQGGY GQSGNSVTSG GYGYGTSAAA

       250        260        270        280        290        300
GAGVAAGSYA GAVNRLSSAE AASRVSSNIA AIASGGASAL PSVISNIYSG VVASGVSSNE

       310        320        330        340
ALIQALLELL SALVHVLSSA SIGNVSSVGV DSTLNVVQDS VGQYVG
```

$NT2RepCT^{YF}$

```
        10         20         30         40         50         60
MGHHHHHHMS HTTPWTNPGL AENFMNSFMQ GLSSMPGFTA SQLDDMSTIA QSMVQSIQSL

        70         80         90        100        110        120
AAQGRTSPNK LQALNMAFAS SMAEIAASEE GGGSLSTKTS SIASAMSNAF LQTTGVVNQP

       130        140        150        160        170        180
FINEITQLVS MFAQAGMNDV SAGNSGRGQG GFGQGSGGNA AAAAAAAAAA AAAAGQGGQG

       190        200        210        220        230        240
GFGRQSQGAG SAAAAAAAAA AAAAAGSGQG GFGGQGQGGF GQSGNSVTSG GYGYGTSAAA
```

250 260 270 280 290 300
GAGVAAGSYA GAVNRLSSAE AASRVSSNIA AIASGGASAL PSVISNIYSG VVASGVSSNE

310 320 330 340
ALIQALLELL SALVHVLSSA SIGNVSSVGV DSTLNVVQDS VGQYVG

NT2RepCT[RL]

10 20 30 40 50 60
MGHHHHHHMS HTTPWTNPGL AENFMNSFMQ GLSSMPGFTA SQLDDMSTIA QSMVQSIQSL

70 80 90 100 110 120
AAQGRTSPNK LQALNMAFAS SMAEIAASEE GGGSLSTKTS SIASAMSNAF LQTTGVVNQP

130 140 150 160 170 180
FINEITQLVS MFAQAGMNDV SAGNSG**L**GQG GYGQGSGGNA AAAAAAAAAA AAAAGQGGQG

190 200 210 220 230 240
GYG**L**QSQGAG SAAAAAAAAA AAAAAGSGQG GYGGQGQGGY GQSGNSVTSG GYGYGTSAAA

250 260 270 280 290 300
GAGVAAGSYA GAVNRLSSAE AASRVSSNIA AIASGGASAL PSVISNIYSG VVASGVSSNE

310 320 330 340
ALIQALLELL SALVHVLSSA SIGNVSSVGV DSTLNVVQDS VGQYVG

NT2RepCT[YFRL]

10 20 30 40 50 60
MGHHHHHHMS HTTPWTNPGL AENFMNSFMQ GLSSMPGFTA SQLDDMSTIA QSMVQSIQSL

70 80 90 100 110 120
AAQGRTSPNK LQALNMAFAS SMAEIAASEE GGGSLSTKTS SIASAMSNAF LQTTGVVNQP

130 140 150 160 170 180
FINEITQLVS MFAQAGMNDV SAGNSG**L**GQG G**F**GQGSGGNA AAAAAAAAAA AAAAGQGGQG

190 200 210 220 230 240
G**F**G**L**QSQGAG SAAAAAAAAA AAAAAGSGQG G**F**GGQGQGG**F** GQSGNSVTSG GYGYGTSAAA

250 260 270 280 290 300
GAGVAAGSYA GAVNRLSSAE AASRVSSNIA AIASGGASAL PSVISNIYSG VVASGVSSNE

310 320 330 340
ALIQALLELL SALVHVLSSA SIGNVSSVGV DSTLNVVQDS VGQYVG

NT2Rep

10 20 30 40 50 60
MGHHHHHHMS HTTPWTNPGL AENFMNSFMQ GLSSMPGFTA SQLDDMSTIA QSMVQSIQSL

70 80 90 100 110 120
AAQGRTSPNK LQALNMAFAS SMAEIAASEE GGGSLSTKTS SIASAMSNAF LQTTGVVNQP

130 140 150 160 170 180
FINEITQLVS MFAQAGMNDV SAGNSGRGQG GYGQGSGGNA AAAAAAAAAA AAAAGQGGQG

190 200 210 220
GYGRQSQGAG SAAAAAAAAA AAAAAGSGQG GYGGQGQGGY GQSGS

2RepCT

```
        10         20         30         40         50         60
MGHHHHHHMR NSGRGQGGYG QGSGGNAAAA AAAAAAAAAA AGQGGQGGYG RQSQGAGSAA

        70         80         90        100        110        120
AAAAAAAAAA AAGSGQGGYG GQGQGGYGQS GNSVTSGGYG YGTSAAAGAG VAAGSYAGAV

       130        140        150        160        170        180
NRLSSAEAAS RVSSNIAAIA SGGASALPSV ISNIYSGVVA SGVSSNEALI QALLELLSAL

       190        200        210
VHVLSSASIG NVSSVGVDST LNVVQDSVGQ YVG
```

Protein preparation

All NT2RepCT variants as well as the CTD were expressed and purified as described previously but the CTD was solubilized by sonication instead of lysosome [1,2]. NT2Rep and 2RepCT were expressed and purified like the CTD. NT2RepCT constructs were concentrated to 200-350 mg/ml in 20 mM Tris pH 8 for long-term storage and protein stocks were prepared by dilution to 650 – 750 µM in deionized Water. CTD constructs have been concentrated to 1 mM, NT2Rep and 2RepCT to 200-300 µM in 20 mM Tris pH 8. In all experiments samples have been prepared by dilution of the protein stocks in the respective buffer.

Fluorescence microscopy

For DroProbe imaging, 50 µM of Sodium 3,3′-{[(1,2-diphenylethene-1,2-diyl)bis(4,1-phenylene)]bis(oxy)}bis(propane-1-sulfonate) and 50 µM of the respective spidroin construct were added to buffer in a 18 well µ-Slide (ibidi) and incubated at room temperature for 5 min. Fluorescence microscopy images were acquired using a Nikon Eclipse Ti series inverted microscope (Nikon) equipped with Crest X-light V2 series confocal unit (Nikon), using 395 nm excitation wavelength, 3% laser power and 457 nm emission wavelength. Images were acquired using an Plan Apo 60x oil immersion objective (Nikon) and a Zyla VSC camera (Andor). NIS-Elements Advanced Research 5.02.03 64-bit software (Nikon) was used for image analysis. Samples in 0.5 M KPi pH 8 were prepared in black half-area 96-well polystyrene microplates with a transparent bottom (Corning) and images acquired using a LSM980-Airy microscope (Zeiss) equipped with an Airy detector2, 405 nm excitation wavelength, 2% laser power and a 40x water objective.

Native mass spectrometry

Mass spectra were acquired on a Micromass LCT ToF modified for analysis of intact protein complexes (MS Vision, The Netherlands) equipped with an offline nanospray source. ESI capillaries were purchased from Thermo. The capillary voltage was 1.5 kV, the cone voltage 50 V, and the RF lens 1.5 kV. The pressure in the ion source was maintained at 9.0 mbar. Ion

mobility mass spectra were acquired on a Waters Synapt G2 travelling wave ion mobility mass spectrometer (Waters, UK) equipped with an offline nanospray source. The capillary voltage was 1.5 kV, the source presure was 8 mbar, and the source temperature was 30 °C. The collision energy in the ion trap was 10 V. Wave height and wave velocity were 12 V and 350 m/s in the IMS cell and 10 V and 248 m/s in the transfer. IMS gas was nitrogen with a flow of 30 mL/min. Spectra were visualized using MassLynx 4.1 (Waters, UK).

Thioflavin T assay

Aggregation kinetics were monitored in bulk solution by measuring total ThT fluorescence at an excitation wavelength of 448 nm and an emission wavelength of 485 nm using a SPARK 20M plate reader (Tecan). The bandwidths were set to 5 nm and the gain to 150. All measurements were conducted at 28°C, without agitation. 50 µM protein solutions were prepared with 10 µM ThT in 50 mM KPi (pH 8), 50 mM KPi (pH 5), 500 mM KPi (pH 8) and 500 mM KPi (pH 5.1 – to keep the final pH equal to 50 mM pH 5 buffer conditions after adding protein solutions) in black half-area 96-well polystyrene microplates with a transparent bottom (Corning). The reactant volume of each replicate was 80 µl and the plates were sealed with transparent cover film to avoid evaporation.

Molecular Dynamics Simulations

The initial structure of the linker peptide (Ac-GSGNSVTSGGYGYGTSAAAGAGV-NH_2) was generated in an extended conformation using the TLEAP module of AMBER 18[3] and subjected to a short simulation of 200 ps in vacuum . It was then used to set-up a four-copy peptide system and placed in the centre of a truncated octahedral box whose dimension was fixed by setting a minimum distance of 6 Å between any peptide atom and the box boundaries. The force-field parameters of monohydrogen phosphate ($HPO4^{2-}$) ion with bcc charges was derived using the GAFF2 force-field through the ANTECHAMBER[4] module of AMBER 18. The number of monohydrogen phosphate ions equivalent to 0.5 M (90) and 0.05 M (10) concentration respectively was computed based on the volume (~ 296256 $Å^3$) of the primary simulation box holding the four-peptide system. The program packmol[5] was then used to generate the initial distribution of the phosphate ions within a sphere of radius 30 Å from the centre-of-mass (COM) of the peptides and subsequently solvated with OPC water model[6]. Molecular dynamics simulations were carried out using the PMEMD module of AMBER 18 with ff19SB force-field parameters[7]. Both the systems were energy minimized, heated, equilibrated, and finally simulated for 1 µs each under NPT (300 K and 1 atm) conditions. Hydrogen-mass repartitioning[8] was applied, and the equation of motion was solved with an integration time step of 4 fs. The regulation of thermodynamic variables (temperature and

pressure), calculation of electrostatic interactions and treatment of hydrogen containing bonds were implemented as previously described by Christopher et. al[9].

Supplementary References

1. Andersson, M. *et al.* Carbonic Anhydrase Generates CO2 and H+ That Drive Spider Silk Formation Via Opposite Effects on the Terminal Domains. *PLoS Biol.* **12**, e1001921 (2014).
2. Arndt, T. *et al.* Engineered Spider Silk Proteins for Biomimetic Spinning of Fibers with Toughness Equal to Dragline Silks. *Adv. Funct. Mater.* **32**, 2200986 (2022).
3. D. A. Case et al., AMBER 18. *University of California, San Francisco* (2018).
4. J. Wang, R.M. Wolf, J.W. Caldwell, P.A. Kollman, D.A. Case, Development and testing of a general amber force field. *J Comput Chem*. 25, 1157-74 (2004).
5. L. Martínez, R. Andrade, E.G. Birgin, J.M. Martínez, PACKMOL: a package for building initial configurations for molecular dynamics simulations. *J Comput Chem*. 30, 2157-64 (2009).
6. S. Izadi, R. Anandakrishnan, A.V. Onufriev, Building Water Models: A Different Approach. *J Phys Chem Lett*. 5, 3863-3871 (2014).
7. C. Tian et al., ff19SB: Amino-Acid-Specific Protein Backbone Parameters Trained against Quantum Mechanics Energy Surfaces in Solution. *J Chem Theory Comput*. 16, 528-552 (2020).
8. C. W. Hopkins, S. LeGrand, R.C. Walker, A.E. Roitberg, Long-Time-Step Molecular Dynamics through Hydrogen Mass Repartitioning. *J Chem Theory Comput*. 11, 1864-74 (2015).
9. C. J. Brown, C. S. Verma, D. P. Lane DP, D. Lama, Conformational ordering of intrinsically disordered peptides for targeting translation initiation. *Biochim Biophys Acta Gen Subj*. 1865, 129775 (2021).

Supplementary Figures

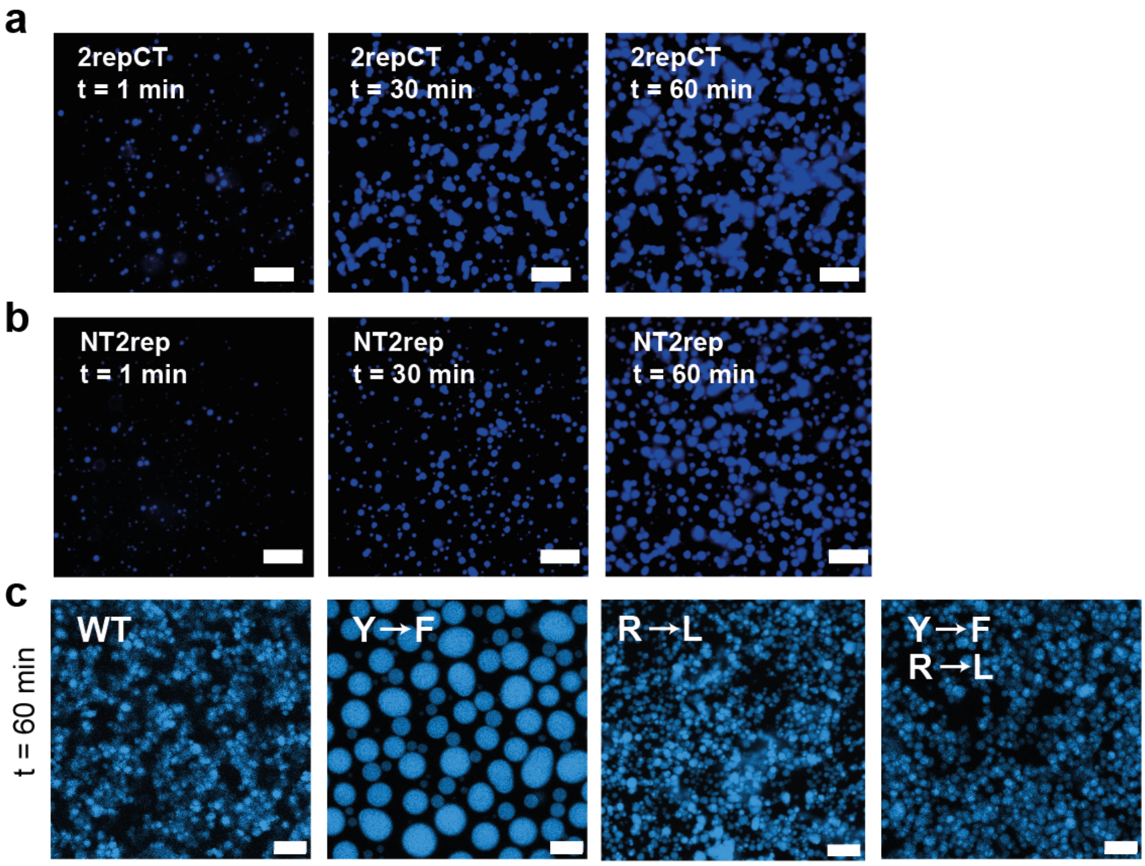

Figure S1. DroProbe images of LLPS and end points of the NT2RepCT variants. (a) Droplet formation of 2RepCT (lacking the NTD) and (b) of NT2Rep (lacking the CTD) show that the CTD increases droplet formation at earlier time points. (c) Droplet formation end points for the NT2RepCT variants shown in Figure 1c. Scale bars are 25 µm (a and b) and 10 µM (c).

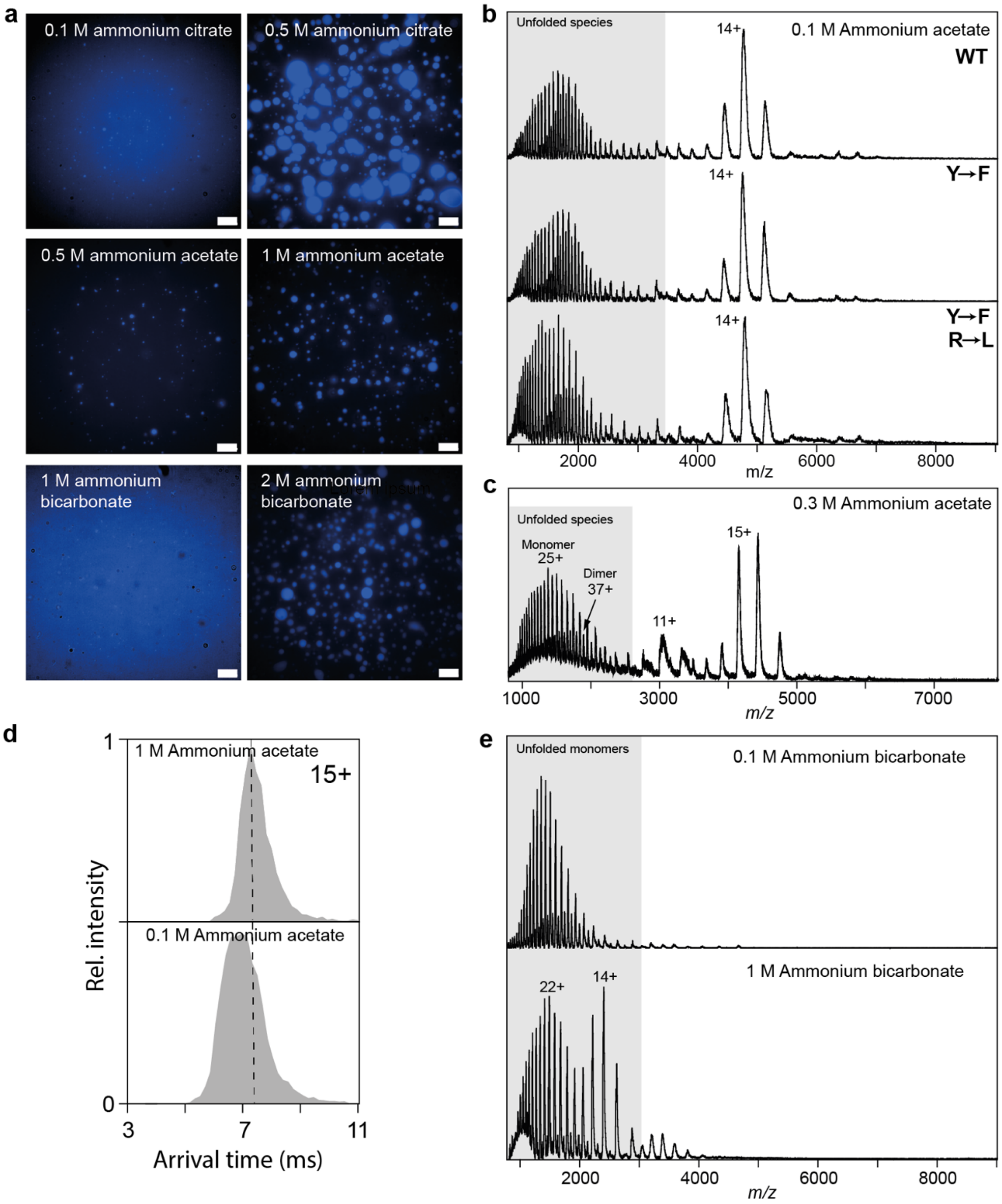

Figure S2. LLPS and native MS of NT2RepCT variants. (a) DroProbe images of freshly dissolved NT2RepCT (Y to F) show the onset of droplet formation between 0.1 and 0.5 M ammonium citrate (top), around 0.5 M ammonium acetate (middle), and between 1 M and 2 M ammonium bicarbonate (bottom). Scale bars are 25 µM. (b) Native MS of wild-type NT2RepCT (top), Y to F (middle) and Y to F + R to L (bottom) in 0.1 M ammonium acetate. No significant differences can be observed regarding the distribution of compact dimers around the 14+ charge state, and unfolded monomer/dimer mixtures (grey area). (c) Mass spectrum of NT2RepCT (Y to F) in 0.3 M ammonium acetate shows an intermediate distribution of monomers and dimers compared to the spectra in 0.1 M and 1 M (see main Figure 2b). (d) The arrival time distribution for the 15+ charge state of the NT2RepCT dimer shows a similar increase in 1 M ammonium acetate compared to 0.1 M as observed for the 14+ dimer (see main Figure 2b). (e) Mass spectra of NT2RepCT (Y to F) in 1 M ammonium bicarbonate reveals complete unfolding and monomerization.

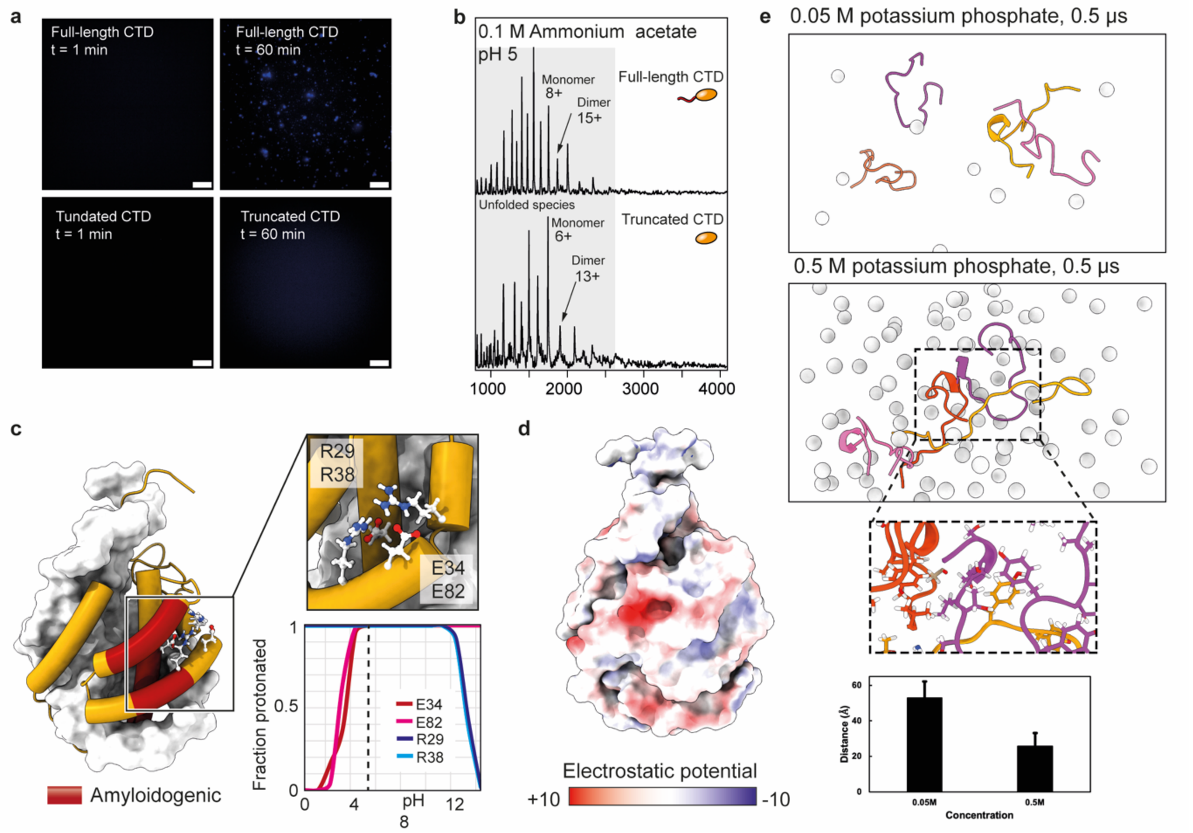

Figure S3. Structural features of the CTD. (a) DroProbe analysis shows that the isolated full-length CTD forms small aggregates in 0.5 M potassium phosphate within 60 minutes, whereas the truncated CTD does not. Scale bars are 25 μM. (b) Native mass spectra of full-length CTD (top) and truncated CT (bottom) at pH 5 show a shift to higher charge states, indicating partial unfolding, but no difference in the monomer/dimer ratio. (c) The conserved charge cluster at the core of the CTD connects two amyloidogenic regions (shown in red) PDB ID 2MFZ. One protomer is rendered as pipes, the other as grey surface. The acidic residues are predicted to become partially protonated as the pH is lowered to 5 (dashed line). (d) Electrostatic surface representation of the CTD dimer shown in panel c. (e) Representative snapshots from all-atom MD simulations of four copies of the amyloidogenic region of the linker in 0.05 M (top panel) and 0.5 M phosphate (middle panel). At high phosphate concentration, the peptides associate into small oligomers driven mainly via hydrophobic interactions and tyrosine stacking (bottom panel). The average distances between the combined centers of mass of the linker peptides are shown below.

Movie S1. Light microscopy of NT2RepCT^{YF} droplets after 30 min in 0.5 M potassium phosphate shows individual droplet fusion events.
